# Immunization of children with inflammatory bowel disease against SARS-CoV-19 infection: A prospective single centre cohort study

**DOI:** 10.1016/j.jvacx.2023.100374

**Published:** 2023-08-19

**Authors:** Dóra Dohos, Anna Karoliny, Eszter Gombos, Éva Rimanóczy, Katalin E. Müller

**Affiliations:** a Institute for Translational Medicine, Medical School, University of Pécs, Pécs, Hungary; b Heim Pál National Pediatric Institute, Budapest, Hungary; c Department of Family Care Methodology, Faculty of Health Science, Semmelweis University, Budapest, Hungary

**Keywords:** IBD, Children, Immunization, SARS-CoV2 immunization, COVID-19 infection

## Abstract

**Background:**

Real-world data of children with inflammatory bowel disease (IBD) after SARS-CoV-2 vaccination are needed.

**Method:**

This prospective, observational study evaluate antibody kinetics of children with IBD 6 months after immunization with COVID-19 mRNA vaccine.

**Results:**

24 children with IBD were included, 22 received immunosuppressive treatment. After five weeks the spike protein antibody level was positive in 95% of the cases. After six months all participants had seropositivity results, though the titre was decreasing.

**Conclusion:**

These data show the effectiveness of SARS-CoV2 immunization and the antibody decay over time, that highlight the importance of booster vaccines.

## Introduction

Patients with inflammatory bowel disease (IBD) have an increased risk for infections due to the characteristics of the disease and the immunosuppressive treatment, thus immunization against vaccine preventable illnesses is crucial in this population. [1], [2] However, patients with IBD may have impaired response to immunization due to their compromised immune system. In an effort to decrease the burden of the pandemic and mitigate the virus, COVID-19 vaccines have been successfully developed. Concerns have been raised that patients with IBD may have impaired serological response or may experience unexpected adverse events after COVID-19 vaccination. [3] However, children with IBD were excluded from the SARS-CoV-2 vaccine clinical trials, so it is crucial to document real-world data of their response to immunization.

The aim of this prospective, single center follow-up study was to assess the safety and efficacy of Comirnaty (BNT162b2, Pfizer-BioNTech; Pfizer, New York, NY) vaccine in children with IBD and to evaluate the antibody kinetics 6 months after immunization.

## Method

After the Hungarian Government has allowed the underaged population vaccination against the SARS-CoV-2, a campaign has been organized for pediatric patients with chronic condition. We consecutively included patients with IBD under 18 years who received two doses of BioNTech, Pfizer vaccine three weeks apart between May 2021 and August 2021.

Blood samples were collected at the time of the first vaccination, then five weeks, three and six months later. At the time of the first immunization, nucleocapsid and spike protein IgG antibodies were measured. After immunizations anti-spike protein IgG antibodies were detected. The Roche Elecsys Anti-SARS-CoV-2 spike immunoassay (range 0.4–250 IU/mL, interpretation: non-reactive < 0.8 IU/mL, reactive ≥ 0.8 IU/mL) and the nucleocapsid immunoassay was applied (reactive cut off index (COI) ≥ 1, non-reactive COI < 1). [4] Cut off for seroconversion was considered anti-spike IgG higher than 0.8 IU/mL. (Supplementary figure 1).

## Results

A total of 24 children from ages 13–18 years were included in this study. Fifteen patients had Crohn’s disease, eight had ulcerative colitis and one had inflammatory bowel disease-unclassified. At the time of the vaccination three patients (13%) received combination therapy (anti-TNF-α treatment and azathioprine), 19 (79%) patients were on monotherapy, eight on azathioprine and eleven on anti-TNF-α therapy, while two (8%) patients did not receive any immunosuppressive treatment. All participants were in clinical remission. (Supplementary table 1).

Based on the patient history, 11 (46%) of the 24 children had PCR confirmed SARS-CoV-2 infection before the start of the immunization process. The nucleocapsid antibody was positive only in six patients (55%). In three more cases the laboratory anti-nucleocapsid test proved previous infection. (Supplementary figure 1, Supplementary table 2).

Five weeks after the first vaccination (two weeks after the second dose), the spike protein antibody level was positive in 95% of the cases (20/21). All results were above the upper-level limit of our test (>250 IU/mL). Seroconversion was not detected in one patient (5%), treated with adalimumab monotherapy. (Table 1, Supplementary figure 1, Supplementary table 2).

**Table 1:** Results of seroconversion in patients with Crohn’s disease and ulcerative colitis.

**PATIENTS**	Seroconversion 5 weeks after the 1st vaccination	Seroconversion 3 months after the 1st vaccination	Seroconversion 6 months after the 1st vaccination
Children with CD	13/14 (93%)	15/15 (100%)	11/11 (100%)
Children with UC/ IBD-U	7/7 (100%)	8/8 (100%)	7/7 (100%)
Total	20/21 (95%)	23/23 (100%)	18/18 (100%)

CD: Crohn’s disease; UC: Ulcerative colitis; IBD-U: Inflammatory bowel disease unclassified.

Three months after the first immunization, the antibody level was positive in all cases (23/23). The patient who did not respond at week 5, also had seroconversion. Furthermore, the antibody level remained above the laboratory threshold limit in 91% (21/23). (Table 1, Supplementary figure 1, Supplementary table 2).

Six months after the first vaccination all participants (18/18) had still seropositive results of anti-spike IgG. The antibody levels remained above the threshold of the laboratory measurement (>250 IU/mL) in twelve patients (67%), while in six cases (33%) the level decreased (range 92.8–247.3 IU/mL). (Table 1, Supplementary figure 1, Supplementary table 2).

One week after the immunization phone interviews were performed with the participants and their legal guardians to gain information about any post-vaccination effects. Serious adverse events were not detected, but eleven from the 24 children (46%) had local reactions and pain, four from the 24 (17%) reported fatigue, one child had a minor headache.

During the study period one child had COVID-19 infection with mild symptoms before and three more children had infection after the 6th month of follow-up.

## Discussion

Our results are in line with the literature in which the Comirnaty vaccine is tolerable and effective despite the chronic inflammatory disease. [3], [5], [6], [7], [8] At the end of the follow-up, all patients had measurable anti-spike antibody level regardless of management of their underlying IBD.

However, we detected decreasing anti-spike antibody levels over time, but in all cases, it remained above the laboratory threshold limit during the follow-up. In the study of Kennedy *et al.,* adequate serological response with decaying antibody titer was detected in adult patients with IBD. [9] Similarly, Quan *et al.,* also experienced decreasing antibody titer during after three doses of vaccination. [5] The importance of booster immunization was proved with the higher antibody titer after a third dose of vaccine compared to the titers after the second injection. [6], [10] Furthermore, Quan *et al.,* evaluated significant antibody responses after the 4th dose of the vaccine. [11] Additionally, comparable antibody concentrations were measured in pediatric IBD patients and healthy controls [7], [8], [12].

Our study has several strengths and limitations. As far our prospective, 6-months follow-up study is the first real-world information about the immunization of pediatric IBD patients against COVID-19. The limitations of the study suggest careful interpretation of the results. The sample size was small, and the follow-up of the whole group was not feasible. The applied antibody kit has a limit of antibody detection, thus the exact antibody concentration is not available.

In conclusion, our real-world data provide clinical relevance, as patients with chronic immune mediated disease are rarely involved in randomized clinical trials dealing with SARS-CoV-2. The results are in line with the literature and confirmed that Comirnaty, SARS-CoV-2 mRNA vaccine is effective and tolerable in children with IBD. These data show the antibody decay over time, which highlight the importance of further booster vaccines and proper vaccination schedules to maintain sufficient humoral protection.

## CRediT authorship contribution statement

**Dóra Dohos:** Formal analysis, Methodology, Project administration, Visualization, Writing – original draft. **Anna Karoliny:** Conceptualization, Data curation, Formal analysis, Investigation, Methodology, Resources, Software, Supervision, Writing – original draft. **Eszter Gombos:** Data curation, Investigation, Validation, Writing – review & editing. **Éva Rimanóczy:** Conceptualization, Investigation, Methodology, Resources, Validation, Writing – review & editing. **Katalin E. Müller:** Conceptualization, Data curation, Formal analysis, Methodology, Project administration, Supervision, Writing – review & editing.

## Declaration of Competing Interest

The authors declare that they have no known competing financial interests or personal relationships that could have appeared to influence the work reported in this paper.

## Data Availability

Data will be made available on request.
