# Supplementary material for: Immunization of children with inflammatory bowel disease against SARS-CoV-19 infection: A prospective single centre cohort study
Source: Vaccine X. 2023 Aug 19;15:100374. doi: 10.1016/j.jvacx.2023.100374 (PMC10562902; doi:10.1016/j.jvacx.2023.100374)
Supplement: Supplementary data 1 [file mmc1.docx]

**Supplementary figure 1** Flowchart of the study periods


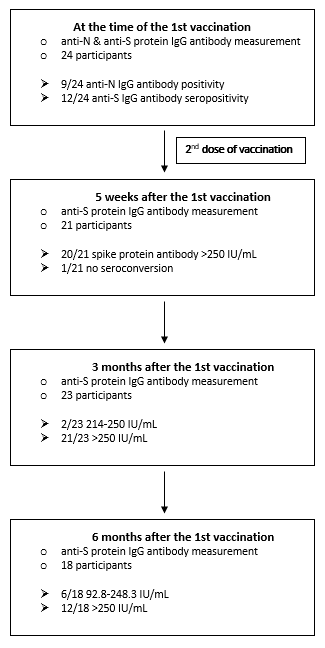


*anti-N: anti-nucleocapsid; anti-S: anti-spike*

**Supplementary table 1** Baseline characteristics of patients

| **characteristics** | **adolescents’**  **population (n=24)** |
| --- | --- |
| male/female | 10/14 |
| age (mean±SD; years) | 16.2±1.5 |
| Crohn’s disease/ ulcerative colitis/ IBD-U | 15/8/1 |
| combination therapy (%)  azathioprine monotherapy (%)  biological monotherapy (%)  5-aminosalycilic acid monotherapy (%) | 13  33  46  8 |

*IBD-U: inflammatory bowel disease unclassified*

**Supplementary table 2** Detailed results of anti-N and anti-S antibodies

|  | *anti-N antibody levels at the time of the 1st vaccination (COI)* | *anti-S antibody levels at the time of the 1st vaccination (IU/mL)* | *anti-S antibody levels 5 weeks after the 1st vaccination (IU/mL)* | *anti-S antibody levels 3 months after the 1st vaccination (IU/mL)* | *anti-S antibody levels 6 months after the 1st vaccination (IU/mL)* |
| --- | --- | --- | --- | --- | --- |
| *patient #1* | 0.362 | 3.5 | >250 | >250 | 120.7 |
| *patient #2* | negative | 1.02 | <0.4 | >250 | 247.3 |
| *patient #3* | negative | <0.4 | >250 | >250 | >250 |
| *patient #4* | negative | <0.4 | >250 | >250 | >250 |
| *patient #5* | 30.62 | >250 | >250 | >250 | >250 |
| *patient #6* | negative | <0.4 | >250 | >250 | 92.7 |
| *patient #7* | 59.48 | >250 | - | - | - |
| *patient #8* | 21.4 | >250 | >250 | >250 | - |
| *patient #9* | 0.37 | 12.5 | >250 | >250 | >250 |
| *patient #10* | negative | <0.4 | >250 | 217.5 | >250 |
| *patient #11* | negative | <0.5 | >250 | 214.2 | - |
| *patient #12* | 0.3 | 1.4 | >250 | >250 | 96.1 |
| *patient #13* | negative | <0.4 | - | >250 | >250 |
| *patient #14* | 98.8 | 45.2 | - | >250 | - |
| *patient #15* | negative | - | >250 | >250 | 122.7 |
| *patient #16* | negative | <0.4 | >250 | >250 | >250 |
| *patient #17* | - | 1.9 | >250 | >250 | 192.7 |
| *patient #18* | 0.278 | - | >250 | >250 | >250 |
| *patient #19* | negative | <0.4 | >250 | >250 | - |
| *patient #20* | 18.54 | 224 | >250 | >250 | >250 |
| *patient #21* | negative | <0.4 | >250 | >250 | >250 |
| *patient #22* | 7.91 | 74.8 | >250 | >250 | - |
| *patient #23* | 0.583 | 3.2 | >250 | >250 | >250 |
| *patient #24* | negative | <0.4 | >250 | >250 | >250 |

anti-N: anti-nucleocaspid; COI: cut off index; anti-S: anit-spike
